# The Accessibility of the Cell Wall in Scots Pine (*Pinus sylvestris* L.) Sapwood to Colloidal Fe_3_O_4_ Nanoparticles

**DOI:** 10.1021/acsomega.1c03204

**Published:** 2021-08-10

**Authors:** Edita Garskaite, Sarah L. Stoll, Fredrik Forsberg, Henrik Lycksam, Zivile Stankeviciute, Aivaras Kareiva, Alberto Quintana, Christopher J. Jensen, Kai Liu, Dick Sandberg

**Affiliations:** † Wood Science and Engineering, Department of Engineering Sciences and Mathematics, Luleå University of Technology, Forskargatan 1, SE-931 87 Skellefteå, Sweden; ‡ Chemistry Department, Georgetown University, 37th and O Streets NW, Washington, D.C. 20057, United States; § Fluid and Experimental Mechanics, Department of Engineering Sciences and Mathematics, Luleå University of Technology, SE-971 87 Luleå, Sweden; ∥ Institute of Chemistry, Faculty of Chemistry and Geosciences, Vilnius University, Naugarduko 24, Vilnius LT-03225, Lithuania; ⊥ Physics Department, Georgetown University, 37th and O Streets NW, Washington, D.C., 20057, United States

## Abstract

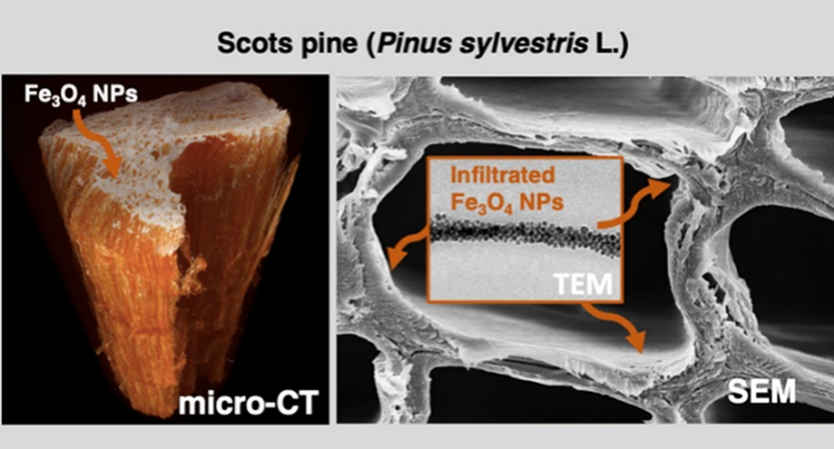

This work presents a rapid and facile way to access the cell wall of wood with magnetic nanoparticles (NPs), providing insights into a method of wood modification to prepare hybrid bio-based functional materials. Diffusion-driven infiltration into Scots pine (*Pinus sylvestris* L.) sapwood was achieved using colloidal Fe_3_O_4_ nanoparticles. Optical microscopy, scanning electron microscopy/energy-dispersive X-ray spectroscopy, transmission electron microscopy, and X-ray powder diffraction analyses were used to detect and assess the accessibility of the cell wall to Fe_3_O_4_. The structural changes, filling of tracheids (cell lumina), and NP infiltration depth were further evaluated by performing X-ray microcomputed tomography analysis. Fourier transform infrared spectroscopy was used to assess the chemical changes in Scots pine induced by the interaction of the wood with the solvent. The thermal stability of Fe_3_O_4_-modified wood was studied by thermogravimetric analysis. Successful infiltration of the Fe_3_O_4_ NPs was confirmed by measuring the magnetic properties of cross-sectioned layers of the modified wood. The results indicate the feasibility of creating multiple functionalities that may lead to many future applications, including structural nanomaterials with desirable thermal properties, magnetic devices, and sensors.

## Introduction

Forests are vital for the environment, society, and economy, and the development of wood products by enhancing their physicochemical properties is of fundamental importance. At the same time, there is an increasing demand for a broad spectrum of cost-effective functional materials with controllable properties,^1,2^ and the synergy between wood and functional materials could provide new multidimensional and multifunctional structures for sustainable production and consumption.^3,4^

The properties of materials are size-dependent, so that when particles are reduced to a nanoscale (1–100 nm), the chemical reactivity, electrical conductivity, optical emissivity, and magnetic permeability change. These size-dependent properties can be precisely tuned by controlling the composition, size, and surface of the nanomaterial.^2,5−7^ Studies show that wood as a matrix with a hierarchical and porous structure offers an exciting platform for the incorporation of nanosized materials.^8^ For example, Trey *et al*. prepared ferromagnetic wood by direct impregnation and ion exchange,^9^ while Merk *et al*. prepared nanobiocomposites with pronounced anisotropic magnetic properties when iron oxide nanoparticles (NPs) were embedded within a wood matrix via *in situ* coprecipitation synthesis,^10^ and Lou *et al*. demonstrated electromagnetic wave-absorbing properties of magnetic wood in which Fe_3_O_4_ was synthesized *in situ* through coprecipitation.^11^ Fe_3_O_4_-wood composites were also prepared by a hydrothermal method,^12^ and Segmehl *et al*. prepared a hybrid iron oxide-wood composite via microwave-assisted thermal decomposition.^13^ In another study, the same group demonstrated infiltration of europium-doped HfO_2_ nanoparticles with a size of 3 nm into Norway spruce (*Picea abies* (L.) Karst.) wood cells.^14^ Gold NPs have also been grown in poplar wood following immobilization of enzymes for heterogeneous biocatalysis.^15^ Attempts have also been made to produce transparent functionalized wood. For example, Gan *et al*. demonstrated the immobilization of Fe_3_O_4_ NPs in delignified wood,^16^ and in another study, transparent and luminescent wood composites were produced by poly(methyl methacrylate) and γ-Fe_2_O_3_@YVO_4_:Eu^3+^ nanoparticle infiltration.^17^ The preparation of luminescent and hydrophobic wood films as optical lighting materials was also demonstrated.^18^ The incorporation of solid inorganic nanomaterials into the wood matrix can be a way to make the wood matrix firmer, as well as making it able to respond to a variety of stimuli in the surrounding environment. Furthermore, the filling of the wood matrix and cell wall cavities with solid inorganic materials may subsequently reduce the inherent vulnerability of the wood to moisture, low and high temperature, exposure to direct sunlight, or biological attack (mold, fungi, bacteria, or insects), which is an important consideration in assessing the performance of wood products.

The detection and visualization of NPs in a wood matrix are important, as the properties of individual materials and their interactions influence the properties of the final material. X-ray microcomputed tomography (micro-CT) allows the nondestructive, three-dimensional imaging of internal structures and has become a well-received tool for such studies.^19,20^ The technique shares the same fundamental imaging principle as computed tomography (CT)^21−23^ but allows a significantly higher spatial resolution, typically on a micrometer or submicrometer scale.^24^ Micro-CT has been adopted and utilized in wood science, mainly using synchrotron light, for anatomical studies^25−27^ and for in situ studies of the deformation of the cellular structure under external loads.^27,28^ The rapid development of high-resolution laboratory-based micro-CT during the last decade has promoted its use and made the technique more accessible. More recently, Wascher *et al*. used micro-CT to study the impregnation volume, penetration paths, and distribution of melamine resin in beech veneer,^29^ and Koddenberg and Militz used the tool for quantitative studies of the cellular structure in European ash (*Fraxinus excelsior* L.).^30^ In another study, micro-CT was used for anatomical structures of willow (*Salix* sp.) trees and to observe xylem tissue development.^31^ CT is also now widely used in artwork analysis. For example, Re *et al*. used standard CT to detect and locate different kinds of materials on a coarse scale on wooden artworks,^32^ and Stelzner and Million performed anatomical and dendrochronological analyses of archaeological wood employing micro- and submicrometer-CT scanners.^33^ CT, allowing precise qualitative analysis, is used in this work as one of the preferred techniques for the study of wood cell walls.

In the present work, Fe_3_O_4_ NP infiltration into Scots pine sapwood has been used to study wood cell wall accessibility with crystalline nanomaterials. Optical microscopy, FE-SEM/EDS, TEM, and XRD analyses were used to assess the nanoparticle distribution. The structural changes and filling of cell lumina were further estimated by micro-CT analysis, chemical changes in the Scots pine induced by the solvent were assessed by FTIR spectroscopy, and the thermal stability of the Fe_3_O_4_-modified wood was studied by TG analysis. The successful infiltration of Fe_3_O_4_ NPs into the wood matrix was confirmed by measuring the magnetic properties of cross-sectioned layers of the modified wood. The results indicate that solid inorganic Fe_3_O_4_ nanoparticles infiltrated into Scots pine sapwood have a potential for creating bio-based materials with multiple functionalities.

## Results and Discussion

### Morphology Evaluation of Treated Wood

**Figure 1:**
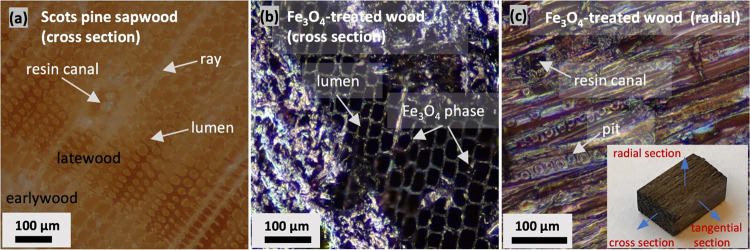
Light microscopic view of (a) cross section of Scots pine sapwood, (b) cross section of the Fe_3_O_4_-treated wood surface, and (c) radial section of the Fe_3_O_4_-treated wood surface. The inset shows the Fe_3_O_4_-treated wood block (10 × 6 × 5 mm) before sectioning.

To evaluate the morphology of the Fe_3_O_4_-treated wood and estimate the homogeneity of nanoparticle coverage, light microscopy was used. An acquired optical image of the neat Scots pine wood shows the microscopic structure of wood in the transverse direction (Figure 1a), where the regions of earlywood (less dense regions with larger lumina) and latewood (denser regions with smaller lumina) are clearly visible. Intercellular resin canals and ray cells can also be seen. These help to accomplish transport of the nutrient liquids in the living tree trunk. In the untreated wood, the cell lumina are lighter in color and are unfilled in both earlywood and latewood.

The surface of the Fe_3_O_4_-treated wood exhibited a different morphology. The gloss of the cell walls and the dark color inside the cell lumina (Figure 1b) indicate that the cell lumina are not saturated with Fe_3_O_4_ but that the NPs homogeneously cover the cell walls. Such a relatively low saturation of the wood structure with Fe_3_O_4_ can be attributed to the initial concentration of NPs in the toluene solvent. Furthermore, the coloration of the modified wood radial surface (Figure 1c) appears homogeneous throughout the entire specimen and confirms that the sedimentation of Fe_3_O_4_ NPs and their adhesion onto the wood was uniform.

**Figure 2:**
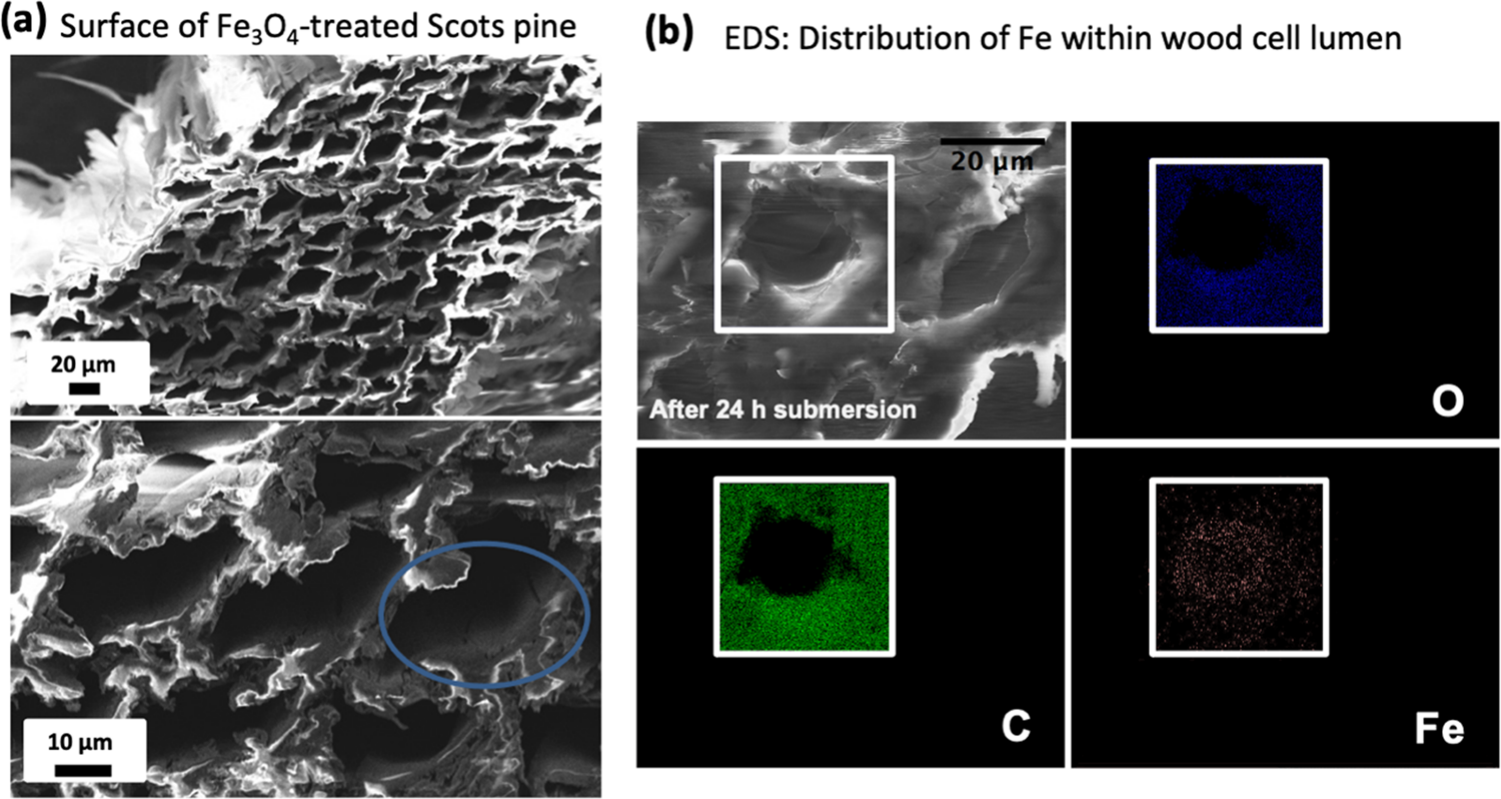
(a) SEM images of tracheid cells in a cross-sectional view of Fe_3_O_4_-modified Scots pine sapwood showing layers of NPs deposited onto the internal parts of cell lumina (marked in a blue circle) and (b) SEM images of the same specimen showing the distribution of individual (C, O, and Fe) elements (designated EDS mapping colors: C – green, O – blue, and Fe – red).

To assess nanoparticle self-diffusion and the distribution of Fe_3_O_4_ within the wood cells, SEM/EDS was used. The cross-sectional SEM images of the interior of the Fe_3_O_4_-treated sapwood block presented in Figure 2a confirm that cell lumina are not filled and that only the cell walls are covered with a layer of solid materials. The EDS-based elemental mapping (C, O, and Fe) further confirms that Fe_3_O_4_ nanoparticles have diffused into the hollow cavities (Figure 2b). It can also be observed that Fe is distributed within the entire wood matrix, i.e., within both the wood cell lumen and the cell wall. This indicates that Fe_3_O_4_ NPs not only attach to the wood cell surface but also diffuse to the intracell and intercell walls, possibly attributed to the solvent effect. It is well-established that the diffusion of solvents into wood polymers (celluloses – linear polysaccharides, hemicelluloses – branched polysaccharides, and lignin – aromatic cross-linked network polymer) depends on the nature of the polymer, the temperature, the concentration of the penetrant, and the shape and size of the diffusing molecule.^34^ Here, two factors have been considered in an attempt to infiltrate Scots pine sapwood with an inorganic solid material: (i) the NP size and dispersibility and (ii) the transport of the solvent through the polymeric wood matrix. Aromatic nonpolar toluene is able to diffuse easily into the polymeric wood matrix,^35,36^ interacting with the wood constituents and able to remove extractive compounds, altering the molecular mobility, the polymer chain relaxation, the connectivity between pores, and subsequently the cellular affinity for the Fe_3_O_4_ nanoparticles within cell walls.^37−41^

**Figure 3:**
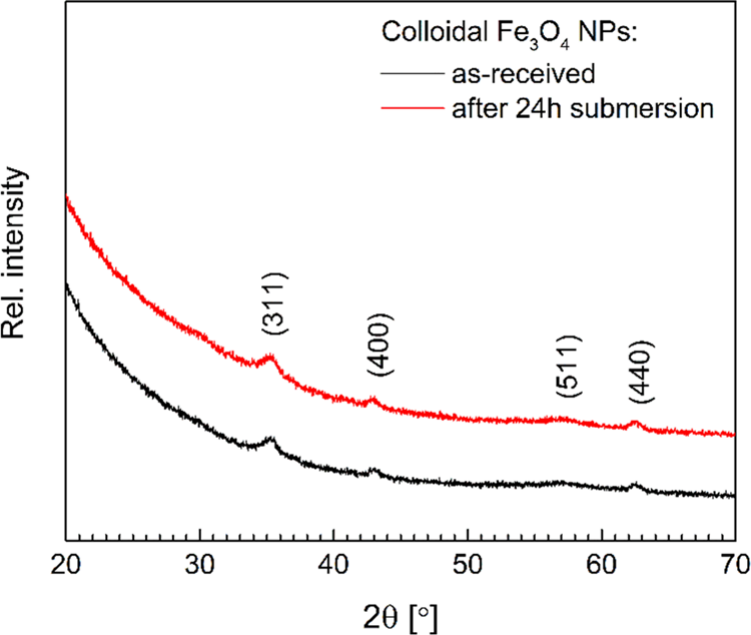
XRD patterns of Fe_3_O_4_ NPs before and after the treatment.

Since toluene prevents aggregation of Fe_3_O_4_ NPs, a stable colloidal solution was maintained during the entire treatment process. XRD patterns (Figure 3) of the colloidal iron oxide before and after the wood treatment confirm the Fe_3_O_4_ phase, with no appreciable evidence of other iron oxide phases (such as Fe_2_O_3_), in agreement with the literature [JCPDS no. 96-900-6190].^42^ The crystallite size of Fe_3_O_4_ before and after the infiltration was estimated from the line broadening in XRD using the Scherrer equation to be 20–25 nm, which confirms that the interaction between the wood components and NPs suspended in toluene did not destabilize the colloidal solution and induce crystallite growth.

**Figure 4:**
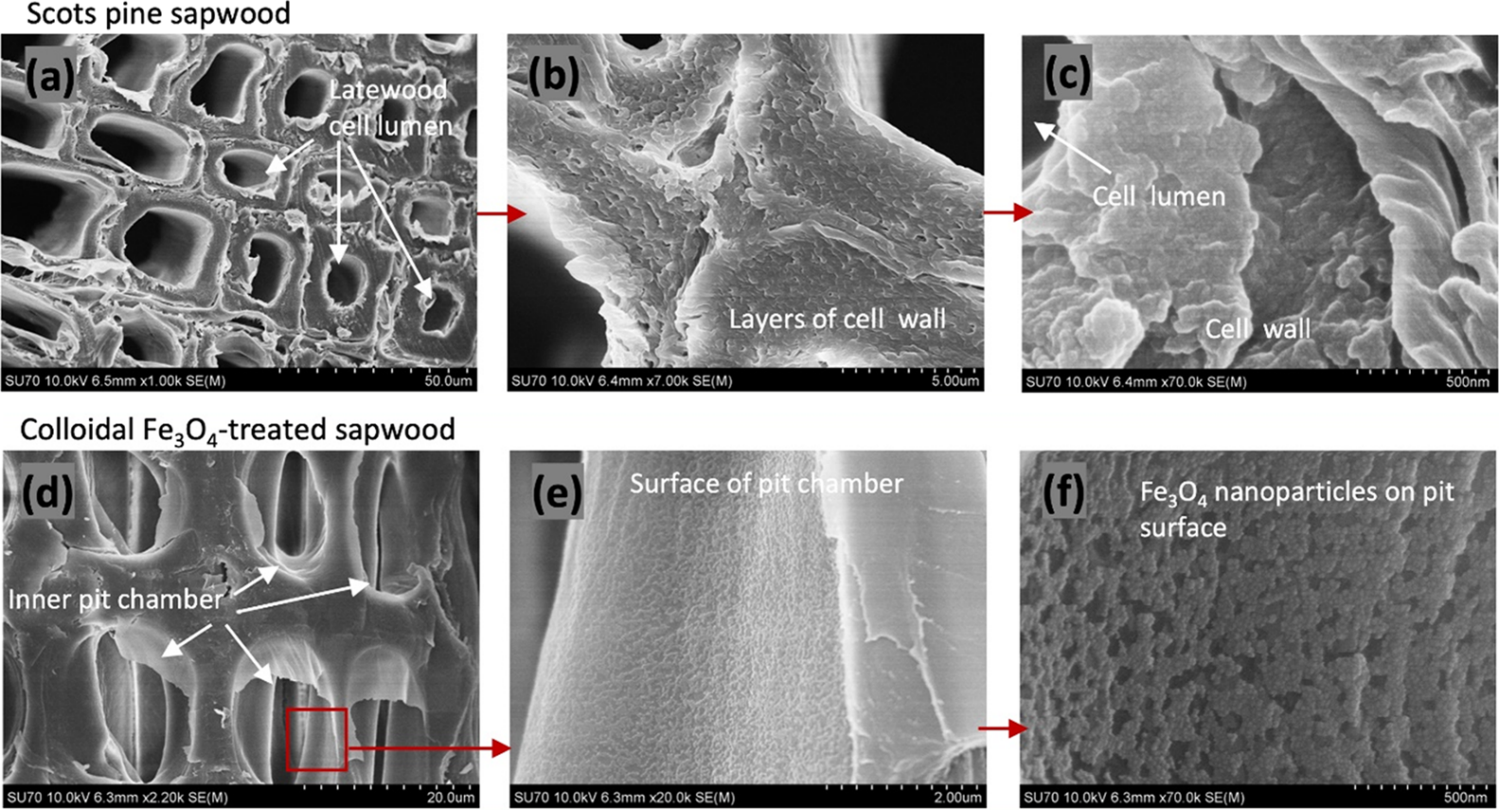
FE-SEM images of (a–c) Scots pine sapwood and colloidal Fe_3_O_4_-treated sapwood shown with progressively increasing magnification: (d) bordered pits, (e) surface of the pit chamber, and (f) Fe_3_O_4_ nanoparticles on the pit surface.

The cross sections of the Fe_3_O_4_-treated wood were also examined to estimate the extent of diffusion of the NPs into the wood, and FE-SEM images are presented in Figure 4. Cross sections of the wood (Figure 4a–c) show a three-layered structure of the cell wall due to the birefringence of the cellulose microfibrils. The untreated cell wall exhibits a relatively smooth morphology (Figure 4c). Different morphological features were observed from the Fe_3_O_4_-treated wood surface, with Figure 4d–f showing the bordered pits of the internal Layer-3 with progressively increasing magnification. The higher-magnification images of the pit membrane show the presence of iron oxide NPs and their uniform distribution on the margo (an impermeable membrane that supports the torus in the bordered pits).^43^ As the pathway to the margo is blocked upon pit closure due to aspiration, the detected NPs indicate that during the treatment, voids and cracks in the wood become saturated with the colloidal solution, which leads to NP sedimentation on the surface of wood tissues. Furthermore, the NPs were uniformly distributed over the entire surface of the wood tissue as clustered aggregates (Figure 4e,f). Magnetic NPs possess high surface energies (large surface to volume ratios), and they tend to aggregate to minimize the surface energy, which likely leads to their clustered deposition on the cell walls. Coverage of the different wood layers varied, however (Figure S1). This can be attributed to the complexity of the wood structure and different NP diffusion pathways within the wood tissue.^14,44^ Examination of deeper layers revealed that cell lumina are not filled (Figure S1). Moreover, toluene has a destructive effect on the wood matrix as it can cause shrinkage and separation between cell walls, and in this study, toluene was used solely to assist magnetic NP infiltration. Therefore, environmentally friendly solvents and alternative wood modification routes should be considered in future work, such as encapsulation of the NPs,^45^ which may allow functional materials to establish good affinity with wood constituencies and form stable bonding.

**Figure 5:**
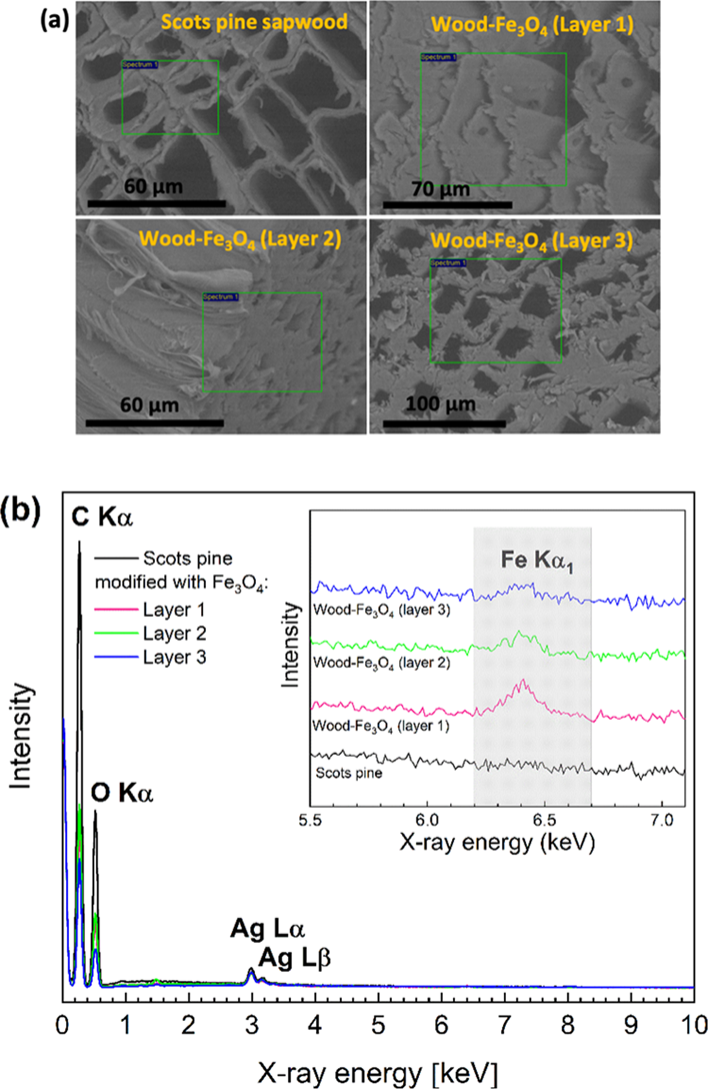
(a) SEM images of untreated wood and wood treated with colloidal Fe_3_O_4_ and (b) EDS spectra of the corresponding layers studied (layer numbers increasing with increasing depth in the wood block). Ag is due to coating. The first SEM image and spectrum (*n* = 3) of each sample are presented.

The cross-sectional SEM images in Figure 5a show the studied regions, and Figure 5b shows the EDS spectra of the untreated wood and the internal three layers cut from the Fe_3_O_4_-modified wood block. The EDS analysis confirmed the presence of Fe in the internal layers. C, O, Al (from the sample holder), and Ag (from the coating layer deposited to remove charging) were detected for the untreated Scots pine sapwood, while Fe appeared in all the Fe_3_O_4_-treated wood specimens. The EDS signal for Fe correlates with the depth of the NP diffusivity, i.e., the signal decreased for the inner layers. The atomic % ratios of C:O:Fe measured in the samples were 62.66:36.42:0.18 for the outer Layer-1, 65.4:33.31:0.12 for the next Layer-2, and 61.09:38.29:0.05 for the innermost Layer-3, showing that there was a gradient in the concentrations of Fe, which decreased toward the middle of the block.

**Figure 6:**
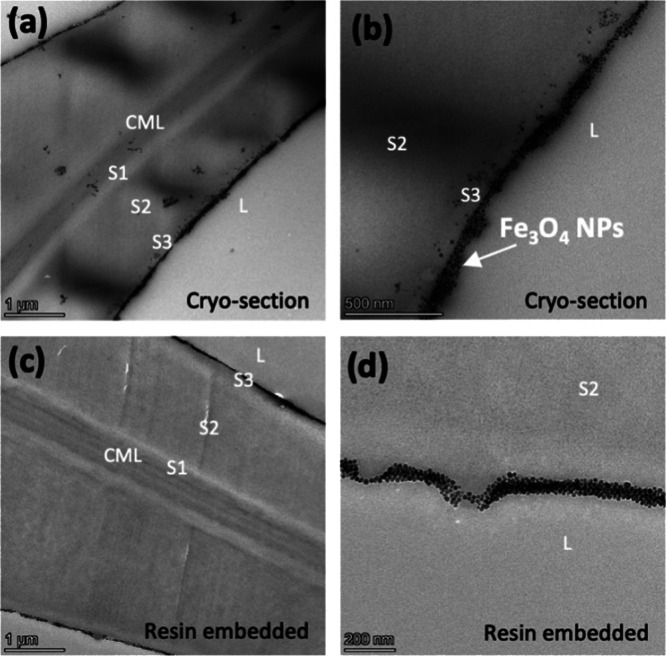
TEM images of Fe_3_O_4_-treated wood: (a,b) cryo-sectioned specimens and (c,d) specimens cut after embedding in the polymer resin (L – lumen; CML – compound middle lamella; S1, S2, and S3 – layers of the secondary cell wall).

The cell wall accessibility to the NPs and their diffusivity depth were further elucidated by TEM analysis examining ultrathin sections of Fe_3_O_4_-treated wood. Figure 6 shows TEM images of the different sections of cell walls, where the compound middle lamella (CML), consisting of the middle lamella and the primary cell wall that connects the cells, as well as a secondary cell wall composed of three layers (S1, S2, and S3),^46^ can be distinguished. The Fe_3_O_4_ NP accumulation on the secondary cell wall S3 layer was observed for all the examined specimens. Although literature reports have identified microcavities in the cell walls in the range of 1.8–80 nm,^47−49^ there was no evidence of the Fe_3_O_4_ NP diffusion into the S2 and S3 layers or the middle lamella region. TEM images of the Fe_3_O_4_-treated wood that was embedded in resin exhibited similar morphological features (Figure 6c,d). The protocol to prepare samples for TEM analysis required them to be treated with solvents (ethanol and water). The Fe_3_O_4_ observed on the cell wall surface indicates that NPs are not washed away when interacting with a liquid but remain compactly adhered to the cell wall surface. However, the amount of F_3_O_4_ NPs adhered to the different parts of the cell wall S3 layer varied. This could be attributed to the reduced concentration of the NPs while the colloidal solution diffuses into the internal layers of the wood block, as well as the complex wood structure that provides different diffusion pathways, i.e., variable length-scale interactions for colloidal NPs with the wood surface. It is also worth mentioning that cracks within cell walls were observed (Figure S2), and this can be attributed to the drying conditions as well as the preparation conditions of the specimens.

### Structural Studies Using Micro-CT

**Figure 7:**
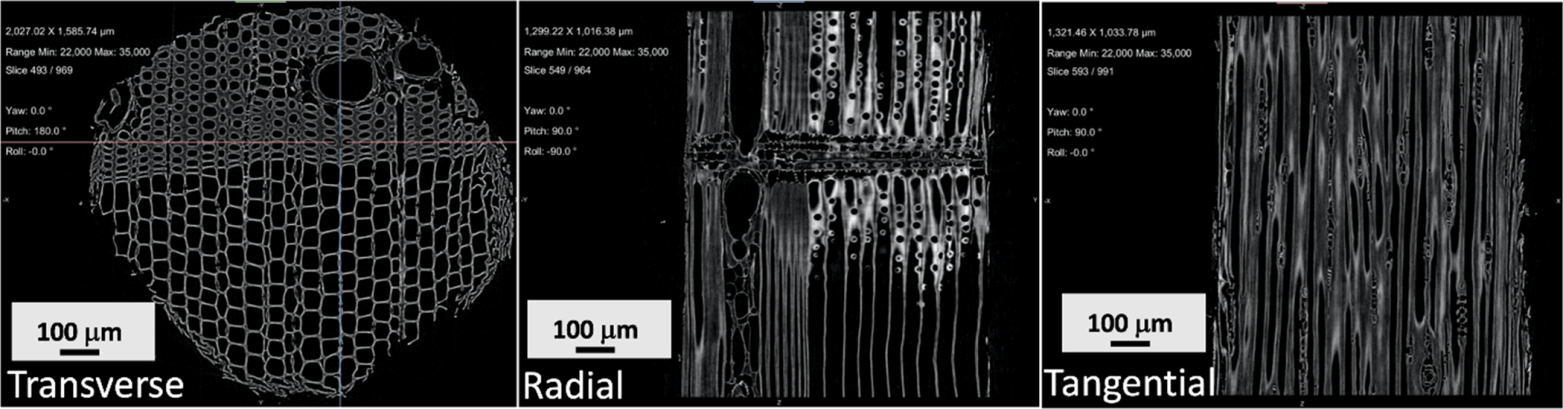
X-ray projections of Fe_3_O_4_-treated Scots pine sapwood in transverse (cross section), radial, and tangential section views.

To confirm that the iron oxide nanoparticles had penetrated into the wood matrix, micro-CT was performed on the Fe_3_O_4_-impregnated wood. Images of the internal Layer-2 of Fe_3_O_4_-treated Scots pine sapwood are presented in Figure 7 and Figure S3. The darker lines observed in the projection images, presented in Figure S3, indicate higher X-ray attenuation and hence structures of higher density. The main part of these structures is believed to reflect the natural variation in wood density that becomes more pronounced along certain directions aligned and orthogonal with the tracheid cell structure. The darkest regions found in some of the dense streaks are believed to indicate diffusion-driven infiltration of NPs into the wood matrix. Similarly, in a recent study, micro-CT measurements were used to study covalent chemical wood modification, i.e., esterification of the wood by applying benzotriazolyl-activated carboxylic acids, and the limitations of voxel contrast and resolution were addressed when interpreting modification of the earlywood zones.^50^ The 2D cross sections from micro-CT data shown in Figure 7 show the wood structure and that the tracheids (cell lumina) are not filled. The grayscale is here reversed (inverted) compared to the projection image in Figure S3, and the level of intensity therefore scales with X-ray attenuation and density. Based on the SEM/EDS data, the CT imaging contrast is probably due to an Fe_3_O_4_ layer present on the lumina cell walls, as well as partial penetration of magnetic NPs into the cell wall cavities. The data also suggests that the self-diffusion of NPs is uniform over the entire specimen but preferential in the transverse direction (Figure S3). Affinity of carboxymethyl cellulose with Fe_3_O_4_ NPs was demonstrated by Anushree *et al*.^51^

**Figure 8:**
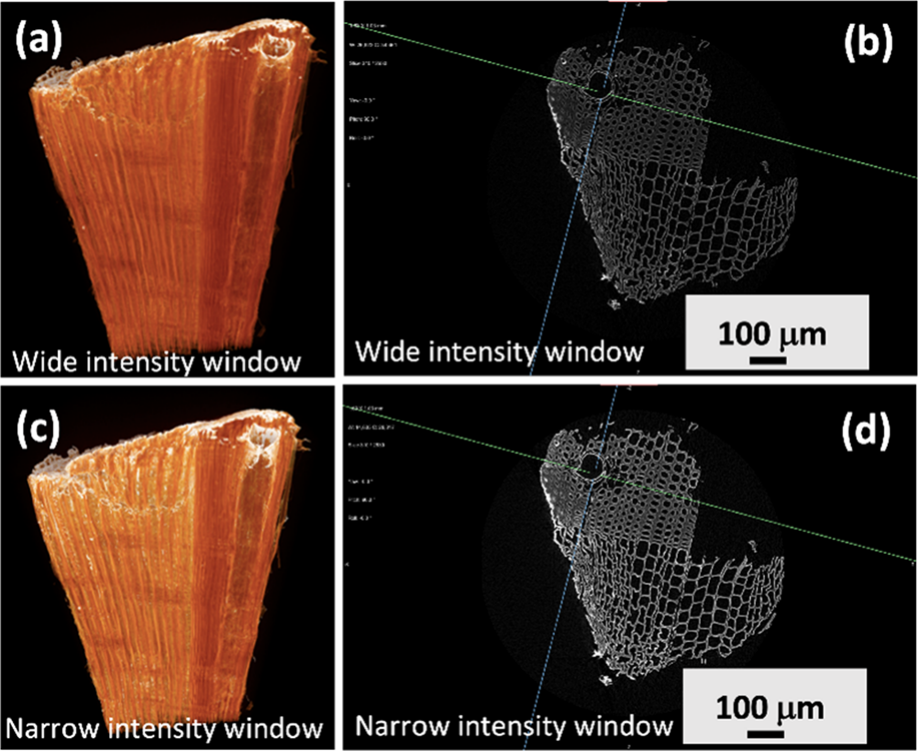
Reconstructed micro-CT 3D images of the Fe_3_O_4_ NP-modified wood and corresponding X-ray projection in the transverse section: (a,b) images using a wider CT number and (c,d) using a narrow CT number.

**Figure 9:**
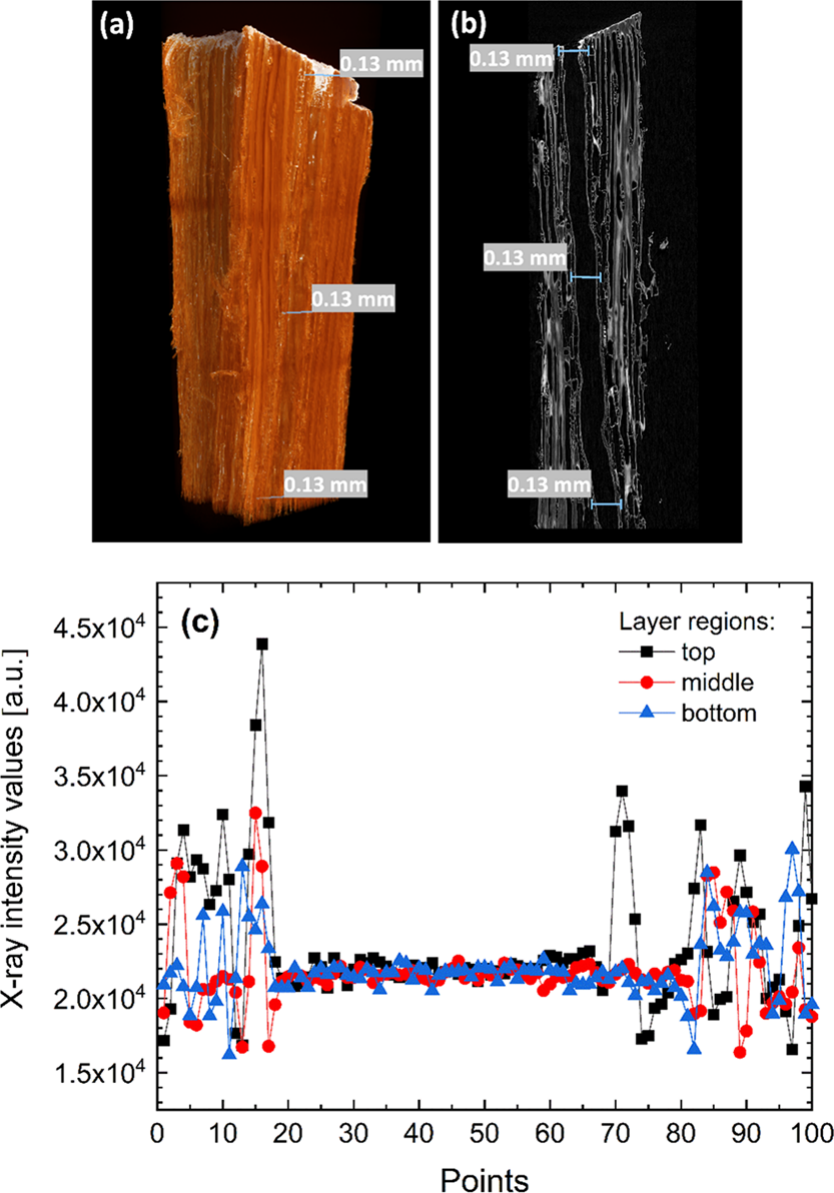
(a) Reconstructed micro-CT 3D image of the Fe_3_O_4_ NP-modified wood showing the regions of measured X-ray intensity profiles, (b) sectioned X-ray projection (radial section), and (c) X-ray intensity profiles of the selected regions within a scanned wood layer.

The diffusivity of Fe_3_O_4_ NPs into the deeper layers of the matrix was further studied by comparing density intensities of anatomical wood structures (Figure S4 shows the region of interest (ROI), the scanned specimen of 2.27 mm height and 1 mm diameter). The reconstructed micro-CT 3D image of the modified wood specimen and the 3D profile of the high-density data (Figure S5) show internal density variations—the brighter regions were observed over the entire specimen with the most intense feature at the material surface. This spatial density inhomogeneity agrees with the previous observations when microscopy (Figures 2 and 5) studies showed the highest accumulation of Fe_3_O_4_ NPs on the wood surface. The reconstructed intensities can be influenced by many factors, such as material porosity, NP diffusion pathways, and artifacts related to tomography, and different intensity windows were therefore used to validate the presence of NPs at different depths of the material. Figure 8 shows reconstructed CT 3D images of the modified wood processed using different intensity windows and corresponding 2D cross sections. In Figure 8a,b, the intensity range is [20,844, 40,951] resulting in a window size of 20,107 units, and in Figure 8c,d, the intensity range is [20,844, 36,628] with a window size of 15,784 units. Additional 2D cross sections in the radial and tangential directions are presented in Figure S6. A brighter attenuation was observed from the surface of the material, as expected (Figure 8a,b). The density spreads equally on the surface between earlywood and latewood but is reduced within more central regions of the layer, as well as slightly more intense from the earlywood compared to the latewood regions. This agrees with the literature reports that earlywood is more susceptible to the different modifications. A narrow intensity window size of 15,784 units resulted in more resolved structures (Figure 8c,d) and confirms the concentration gradient of the precapped Fe_3_O_4_ NPs within the wood matrix. Additional 3D renderings from the micro-CT data are presented in Figure S7, comparing a number of cross sections for the two different intensity windows. The X-ray attenuation intensity profiles of modified wood at the different depths of the sectioned layer were also measured. Selected regions and corresponding depth profiles of the representative projection of the Fe_3_O_4_ NP-modified wood, shown in Figure 9, show that the surface (region at the top) of the specimen exhibits the highest contrast, and only a small variation in intensity was observed between a region in the middle and a region at the bottom. The actual distance of the profiles of the top and middle regions is 123 μm and of the bottom region is 126 μm (Euclidean distances based on the positions of the first and last point for each profile). Thus, although further research, such as synchrotron or NMR studies, might be needed to confirm complete Fe_3_O_4_ nanoparticle intracell diffusivity, the potential to define the material microstructure is clear. Furthermore, with wood structure-penetrating Fe_3_O_4_ NPs, the formation of cracks within the matrix was not observed, and this further indicates that by carefully selecting treatment conditions, different nanosized materials would be able to intercalate intracell and intercell walls. Moreover, micro-CT results clearly showed that by varying the intensity window, more information and more clear data interpretations could be obtained, and this is particularly important in the characterization of the nanostructure of solid hybrid (inorganic–organic) composites.

### Assessment of Chemical Composition via Spectroscopy

**Figure 10:**
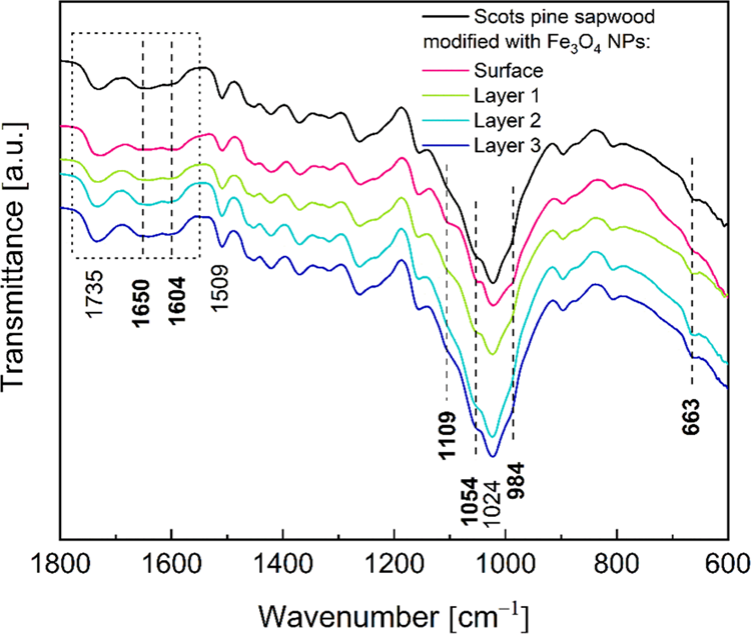
FTIR spectra of pure and different layers of Fe_3_O_4_-treated Scots pine sapwood.

FTIR spectra of the cross-sectioned untreated and Fe_3_O_4_ NP-infiltrated Scots pine sapwood are shown in Figure 10 (full IR spectra are presented in Figure S8). The spectrum from the untreated wood showed typical absorption bands that are assigned to celluloses, hemicelluloses, and lignin,^52^ but in the Fe_3_O_4_ NP-modified wood, of the three regions, only the spectra recorded for the surface exhibited slightly different absorbencies. The bands located in the 2940–2840 cm^–1^ region that originated from the C–H asymmetric stretching in methyl and methylene groups of aliphatic hydrocarbons were more pronounced than in the untreated wood, attributed to the effect of the solvent on the wood constituents and the removal of low-molecular-weight components. In the 1750–1540 cm^–1^ region, the lignin gives characteristic IR absorption bands, and the ratio of the intensity of the band at 1604 cm^–1^ (C=C aromatic skeletal vibrations) to that at 1650 cm^–1^ (C=O stretching vibrations) increased after Fe_3_O_4_ treatment. At lower wavenumbers, such as 1509 (aromatic skeletal vibrations), 1450 (C=C, C–H, and O–H in-plane deformation and −CH_3_ asymmetric bending (lignin)), and 1419 cm^–1^ (C–H aromatic skeletal vibrations (lignin) and −CH_2_ bending deformation (celluloses)), the signals were unchanged, indicating that components are relatively unaffected by the treatment. The fingerprint 1150–950 cm^–1^ region is dominated by bands due to various polysaccharide vibrations.^52^ In the case of the Fe_3_O_4_-treated wood, the bands at 1106, 1054, and 984 cm^–1^ were more pronounced than those of untreated wood and correspond to the C–O–C stretch and O–H from celluloses and hemicelluloses, C–O–C symmetric stretch (celluloses and hemicelluloses), and aromatic C–H out-of-plane deformation (celluloses and hemicelluloses), respectively. This could be clearly attributed to the effect of the solvent on the individual wood components. The band at 663 cm^–1^, assigned to the C–OH out-of-plane bending vibrations of celluloses, also showed a slightly lower absorbance in the spectrum of the treated wood surface.^53^ According to the literature, the Fe–O bond of Fe_3_O_4_ gives a broad band at ca. 600 cm^–1^ (tetrahedral sites of the crystal lattice), which due to oxidation of Fe^2+^ and Fe^3+^ splits into bands at around 561 and 667 cm^–1^.^51^ The latter probably overlaps with the cellulose peak. Nevertheless, the spectra from the cross sections of the Fe_3_O_4_ NP-treated wood were almost identical to those of the untreated wood.

### Thermal Behavior

**Figure 11:**
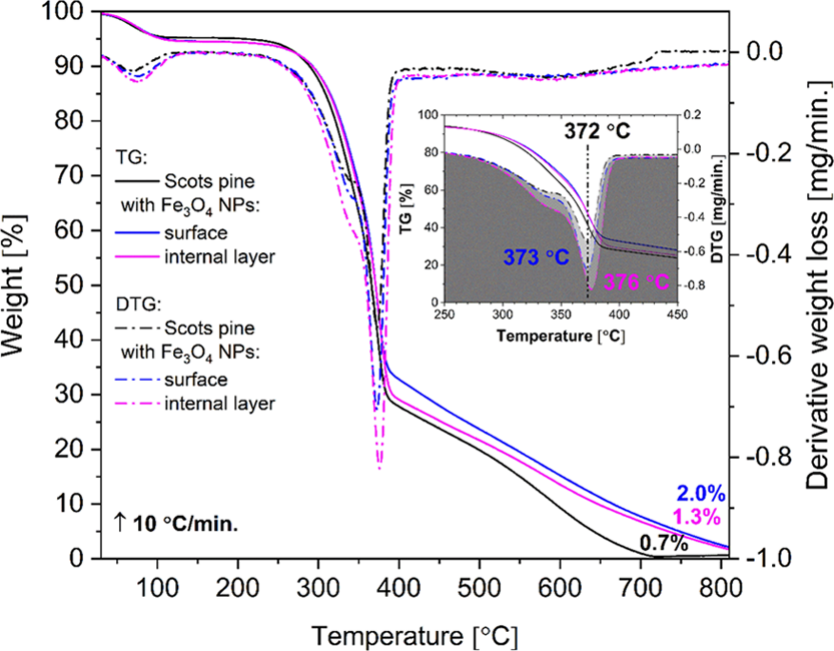
TG/DTG curves of the untreated and treated Fe_3_O_4_ NP Scots pine sapwood.

The thermal behavior of Scots pine sapwood untreated and treated with Fe_3_O_4_ NPs was evaluated by TG analysis. The TG and derivative thermogravimetric (DTG) curves in Figure 11 show three stages of weight loss. The first weight loss of ∼5% for untreated Scots pine and of ∼6% for Fe_3_O_4_ NP-treated wood was observed up to 120 °C and was assigned to the removal of adsorbed water. The second significant weight loss (66% for untreated wood, 60% for the surface layer of Fe_3_O_4_-treated wood, and 64% for the internal layer of the block) was observed between 280 and 410 °C. This weight loss was ascribed to the decomposition of wood components, as hemicelluloses, celluloses, and lignin are pyrolyzed at 200–300, 280–350, and 280–650 °C, respectively.^52^ The DTG curves (Figure 11, inset) show that pyrolysis of the Fe_3_O_4_ NP-treated wood took place over slightly broader temperature regions, at 373 (surface) and 376 °C (internal layer), and a small shift to higher temperatures was observed. This was attributed to the inorganic solid phase present within the wood structure. The final weight loss for the untreated wood was observed at 715 °C, leaving a residue of about 0.7% with no mass change at 810 °C. In the Fe_3_O_4_-treated wood, the surface layer and the internal layer showed a more gradual loss of weight between 390 and 810 °C. The residual masses after the burn-off of the Fe_3_O_4_-treated wood samples increased with increasing iron oxide concentration and were 2 and 1.3% for the surface layer and the internal layer, respectively. The treatment gave a shift of the onset temperature to a higher value and to a mass gain after pyrolysis. A similar mass gain was reported by Tray *et al*. when magnetic Co/MnFe_2_O_4_ was deposited on the cell walls.^9^ This further suggests that using a simple and inexpensive self-infiltration method as well as nanostructured oxide-based materials, the wood modified with inorganic solids could be produced, with resulting properties that depend on the wood and inorganic components, as well as on the interaction of both.

### Magnetic Properties

**Figure 12:**
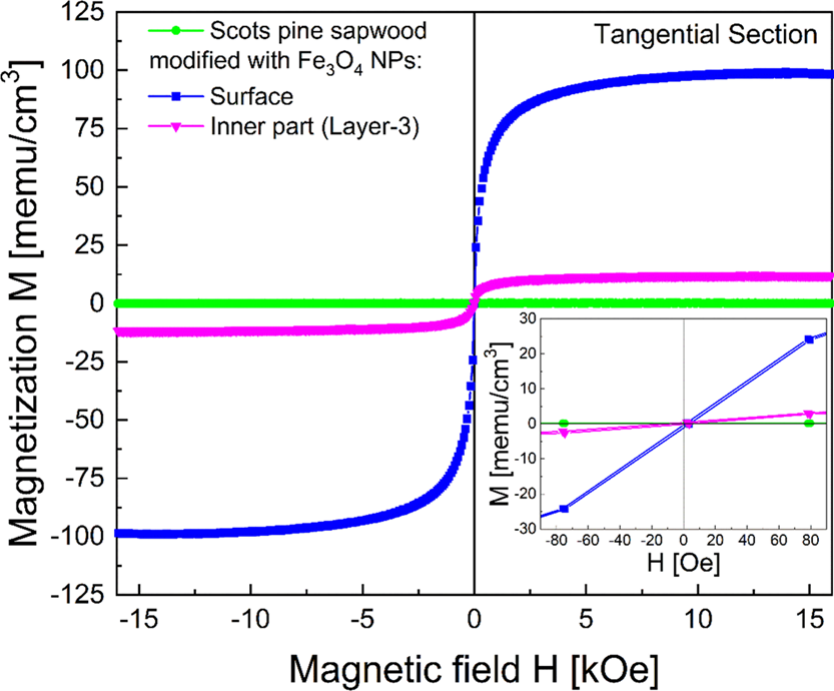
Room temperature *M*–*H* curves of the untreated and Fe_3_O_4_-treated Scots pine sapwood (tangential section). The inset shows a narrow region of the hysteresis loop with diagonal characteristics in the low-field region. Magnetization values are normalized to the wood specimen volume.

The self-diffusion of Fe_3_O_4_ nanoparticles into Scots pine sapwood was further elucidated by measuring the magnetic hysteresis loops at room temperature of the untreated and Fe_3_O_4_-treated wood. The lack of any appreciable magnetic signal for the untreated Scots pine (Figure 12) confirms the paramagnetic nature of wood (flat line). In contrast, the signal arising from the surface region of Fe_3_O_4_-treated wood shows clear, ferromagnetic-like behavior (Fe_3_O_4_ nanoparticles are ferrimagnetic, i.e., there are two opposite spin sublattices with unequal spins).^42^ The inner Layer-3 of the treated wood exhibited a weaker magnetic signal than the surface, suggesting a lower concentration of magnetic nanoparticles in the former. Nevertheless, the results confirm that short-range infiltration of NPs into wood can be easily achieved across the sample thickness by self-diffusion. No appreciable coercivity was observed in any of the specimens (Figure 12, inset) due to the superparamagnetic nature of the ultrasmall nanoparticles.^54−56^ This is in agreement with the literature where similar magnetization behavior was observed in Fe_3_O_4_-carboxymethyl cellulose nanocomposites containing NPs of different sizes.^51^ Similar magnetic curves were obtained from magnetic wood-nanocomposite hybrid materials when ferrite was synthesized within Norway spruce and European beech (*Fagus sylvatica* L.) wood.^10^ Magnetic wood was also studied earlier, where Oka *et al*. investigated three types of magnetic woods, i.e., Mn–Zn ferrite wood composites, and demonstrated that DC magnetic characteristics were dependent on the magnetic material, the density of the magnetic material, and the structure of the wood.^57^

Taking into consideration different treatment processes, variability in the wood structure, and NP properties, overall results indicate that the properties of different wood species could be tailored and modified according to the required application. The development of stable environmentally friendly water-based suspensions shall also be explored in combination with an industrial wood pressure-treatment method to obtain the complete diffusivity into the different wood species of larger sizes.

## Conclusions

Fe_3_O_4_ nanoparticles (NPs) were successfully infiltrated into the Scots pine sapwood matrix in a rapid and facile manner using a colloidal solution of iron oxide in toluene with a particle size of 20 nm. Optical microscopy showed that the treated wood blocks were uniformly covered with an iron oxide layer, and FE-SEM/EDS revealed a uniform distribution of Fe_3_O_4_ NPs on the walls of the cell lumen as well as a decrease in the amount of Fe with increasing depth into wood blocks. TEM analysis confirmed the lumen-sided coating, i.e., Fe_3_O_4_ NP accumulation on the cell wall S3 layer, but no diffusion into the S1 and S2 layers of the cell walls or the middle lamella. Micro-CT analysis confirmed that infiltrated NPs do not accumulate within random cell lumina and that there were no collapsed or filled parenchyma cells in the Fe_3_O_4_ NP-treated wood. CT-analysis also showed that cell walls exhibited very clear signals, indicating the infiltration of precapped NPs into the cell lumina. CT further showed that the phase-contrast signal was uniformly strong through the entire specimen and that using different intensity windows, a stronger phase-contrast signal can be obtained from infiltrated nanoparticles, which shows a potential in characterization of the nanostructural features of solid hybrid (inorganic–organic) composites. FTIR spectroscopy showed that the surface layer of treated wood exhibited a stronger signal from celluloses and lignin, confirming that toluene affects the wood components, whereas the layers of the treated wood showed spectral features similar to those of untreated wood. Thermogravimetry revealed that internal layers of the treated wood had a slightly lower degradation temperature and that when Fe_3_O_4_ NPs were present, the residue after pyrolysis was higher than that of the untreated wood. The measurements of the magnetic properties of cross sections of treated wood confirmed that Fe_3_O_4_ NPs had infiltrated into the Scots pine matrix and that the diffusion of nanoparticles diminished within the internal layers of the wood. Results show that crystalline nanosized materials have a potential in wood cell wall modification and for the development of hybrid bio-based materials with multiple functionalities.

## Experimental Section

### Chemicals and Wood Treatment

Iron oxide(II,III) nanoparticles (20 nm average particle size, 5 mg/mL in toluene; Sigma-Aldrich) were used to treat blocks of Scots pine (*Pinus sylvestris* L.) sapwood with dimensions of 0.5 × 0.5 × 10 mm (tangential (T) × radial (R) × longitudinal (L)) cut from sawn timber. Prior to the chemical treatment, freshly cut wood blocks were kept at room temperature for two weeks and then submerged in vessels of toluene solutions containing colloidal Fe_3_O_4_ nanoparticles, tightly closed with a plastic cover, and kept for 24 h at room temperature (∼23 °C). The Fe_3_O_4_-modified wood blocks were then removed from the suspension and allowed to dry for 24 h at room temperature and cross-sectioned into of 1 mm-thick slices. These slices were numbered in a successive order from the outer side to the center of the wood block as Layer-1, Layer-2, and Layer-3. Modified wood blocks and obtained slices were analyzed without performing washing steps.

### Characterization

Light microscopy was performed on an Olympus DSX1000 digital stereomicroscope coupled with a DSX10-SXLOB Plan 10x/0.20 co/0/OFN22 WD41.1 objective lens, and images were acquired in a dark-field illumination mode. The morphological features of the cross-sectioned specimens were evaluated using a field emission scanning electron microscope (FE-SEM, SU70, Hitachi). The Ag-coated specimens were examined using secondary electrons (SE) and an electron beam acceleration voltage of 10 kV. SEM images were also taken with a Zeiss SUPRA55-VP (at an acceleration voltage of 5 kV with an in-lens detector) equipped with an energy-dispersive X-ray spectrometer, controlled using INCA software (Oxford Instruments). Energy-dispersive X-ray spectroscopy (EDS) analysis and elemental mapping were performed using the secondary electrons (SE) and an electron beam acceleration voltage of 15 kV. A table-top scanning electron microscope (SEM, TM3000, Hitachi, 15.0 kV acceleration voltage) was used to estimate the elemental composition of cross-sectioned layers of the modified wood. An X-ray acquisition time of 120 s was used to obtain the EDS spectra (*n* = 3 for each feature of interest). Transmission electron microscopy (TEM) analysis was performed using a Talos FEI (L 120C) transmission electron microscope. Two sets of specimens were investigated, sections of Fe_3_O_4_-treated wood prepared by cryo sectioning and sections of treated-wood after plastic embedding. In cryo sectioning, samples were submerged in 2.3 M sucrose solution for 12 h and cooled down in liquid nitrogen. Ultrathin sections were cut at −120 °C using a Leica EM UCF7 microtome equipped with a cryo chamber. The sections collected from the sucrose-methylcellulose solution were mounted on copper hexagonal mesh grids. Before staining with uranyl acetate, the mixture of sucrose and uranyl acetate was washed away by short incubation in Milli-Q water. Prior to TEM observation, the samples were embedded in a methylcellulose-uranyl acetate mixture. In plastic embedding, samples were rehydrated in an ethanol series, and solvent concentration was increased gradually from 50% to pure ethanol. After the treatment in pure ethanol (Ethanol Aa 99.7%, Solveco), samples were embedded in ethanol-Spurr (Sigma-Aldrich) resin mixtures of ratios 3:1, 1:1, and 1:3 and in pure resin for 12 h. These samples were then embedded in blocks and incubated further at 65 °C for 12 h to obtain complete polymerization of the resin. Ultrathin sections were cut using a Leica EM UCF7 microtome. The sections were mounted on copper hexagonal mesh grids. Prior to TEM observation, the sections were stained with 5% aqueous solution of uranyl acetate. Infrared spectra were recorded with a Fourier transform infrared (FTIR) spectrometer (Frontier FTIR, PerkinElmer, ZnSe/diamond ATR crystal, DTGS detector, 4000–600 cm^–1^, 4 scans). X-ray powder diffraction (XRD) patterns were obtained using a Rigaku, Ultima IV X-ray diffractometer with Cu Kα radiation at 40 kV and 30 mA and a D/teX silicon strip detector (2θ = 15–70°, 0.02 ss, 1°/min.). The thermal behavior of the neat and Fe_3_O_4_-modified wood was evaluated by thermogravimetry (TG) using a PerkinElmer TGA 4000 instrument. The weight of the specimens was about 6 mg, and they were heated from 30 to 850 °C at a constant rate of 10 °C/min, with nitrogen being used as the purge gas (flow supply of 2 bar).

### X-ray Micro-CT

Samples of Fe_3_O_4_-modified wood were scanned using a Zeiss Xradia 510 Versa (Carl Zeiss X-ray Microscopy, Pleasanton, CA, USA) 3D X-ray microscope (XRM), as shown in Figure 12. This imaging system combines flexibility with high-resolution and high-contrast capabilities.^58^ It has multiple detector objectives enabling sample imaging with a number of pairings of the resolution and the field of view, analogous to a light microscope. The maximum spatial resolution in terms of 10% MTF was 0.7 μm, while the spatial resolution in terms of voxel resolution (minimum voxel size) was 70 nm. The scan was carried out using a 20× objective with a field of view (FOV) of 1.0 mm and a voxel size of 1.0 μm. The X-ray tube voltage and power were 50 kV and 4 W, respectively, and the scan was carried out without any X-ray filters. During the scan, 1601 projection images (radiographs) were acquired, over a sample rotation of 360°, with 4.0 s exposure time, which resulted in a total scan time of 2.5 h.

**Figure 13:**
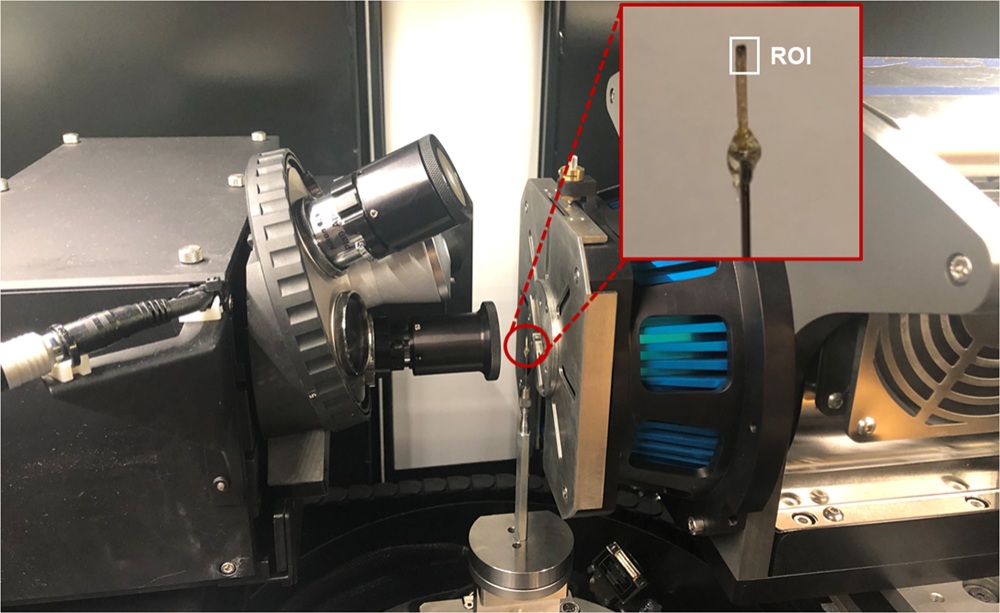
Experimental setup of the micro-CT study, showing a Zeiss Xradia 510 Versa system (left) and a close-up of the Fe_3_O_4_-treated wood sample (right), where the ROI (region of interest) marks the scanned region (photograph courtesy of Dr. Fredrik Forsberg, Luleå University of Technology, Sweden).

Prior to each scan, a 0.5 h “warm-up” scan procedure was carried out with fewer projections and a shorter exposure time, to prevent potential motion artifacts due to thermal expansion from friction. The reconstructed volumes correspond to a cylindrical region with a diameter and height of 1.0 mm, denoted as ROI in Figure 13. The tomographic reconstruction was carried out using filtered back-projection with Zeiss Scout-and-Scan Reconstructor software (version 14.0). The 3D visualization and quantitative analysis of the samples were obtained using Dragonfly Pro software (Object Research Systems, ORS).

### Magnetic Properties

Magnetic hysteresis curves of treated and untreated wood specimens were determined with a vibrating sample magnetometer (Princeton Measurement Corporation MicroMag 3900 Series VSM, USA) and a Magnetic Property Measurement System (MPMS3, Quantum Design). Thin specimens of neat wood and Fe_3_O_4_-treated wood were cut (0.1 cm) before measurement using a ceramic blade.

## Supporting Information Available

SEM images of the Fe_3_O_4_-modified Scots pine sapwood showing unfilled cell lumina and NP distribution in the pits; TEM images of the Fe_3_O_4_-modifed Scots pine showing cracks in the cell walls; image of the specimen used for CT analysis; micro-CT setup showing the region of interest (ROI) and dimensions of the Fe_3_O_4_ NP-modified Scots pine used to study the NP distribution over the entire specimen; reconstructed micro-CT 3D images of the Fe_3_O_4_ NP-modified wood showing the whole scanned specimen that was cut from the internal part of the 1 cm × 0.5 cm × 0.5 cm wood block and high density profile with different distributions of the Fe_3_O_4_ NPs over the entire specimen (bright places); X-ray projections of the modified wood from radial and tangential sections using different intensity windows; reconstructed micro-CT images showing a number of cross sections of the Fe_3_O_4_ NP-modified wood for two different intensity windows; full region of FTIR spectra of Scots pine sapwood and Fe_3_O_4_-modified wood (PDF)

## ABREVIATIONS

NPs: nanoparticles
SEM/EDS: scanning electron microscopy/energy-dispersive X-ray spectroscopy
FE-SEM: field emission scanning electron microscopy
TEM: transmission electron microscopy
XRD: X-ray powder diffraction
micro-CT: microcomputed tomography
FTIR: Fourier transform infrared spectrometry
TG: thermogravimetry
DTG: derivative thermogravimetric
IR: infrared
CML: compound middle lamella
ROI: region of interest
