# Supplementary material for: The Accessibility of the Cell Wall in Scots Pine (Pinus sylvestris L.) Sapwood to Colloidal Fe3O4 Nanoparticles
Source: ACS Omega. 2021 Aug 10;6(33):21719–29. doi: 10.1021/acsomega.1c03204 (PMC8388106; doi:10.1021/acsomega.1c03204)
Supplement: Supplementary file 1 — ao1c03204_si_001.pdf [file ao1c03204_si_001.pdf]

Supporting information

The accessibility of cell wall in Scots pine (*Pinus sylvestris* L.) sapwood to colloidal Fe_3O_4 nanoparticles

Edita Garskaite[*a], Sarah L. Stoll*[b], Fredrik Forsberg*[c], Henrik Lycksam*[c], Zivile Stankeviciute*[d], *Aivaras Kareiva*[d], Alberto Quintana*[e†], Christopher J. Jensen*[e], Kai Liu*[e], Dick Sandberg*[a]

[a]Wood Science and Engineering, Department of Engineering Sciences and Mathematics, Luleå University of Technology, Forskargatan 1, SE-931 87 Skellefteå, Sweden

[b]Chemistry Department, Georgetown University, 37th and O Streets NW, Washington, D.C. 20057, United States

[c]Fluid and Experimental Mechanics, Department of Engineering Sciences and Mathematics, Luleå University of Technology, SE-971 87 Luleå, Sweden

[d]Institute of Chemistry, Faculty of Chemistry and Geosciences, Vilnius University, Naugarduko 24, Vilnius LT-03225, Lithuania

[†] Present Addresses:
Institut de Ciència de Materials de Barcelona (ICMAB-CSIC), Campus UAB, Bellaterra 08193, Catalonia, Spain
Georgetown University, 37th and O streets NW, Washington, D.C. 20057, United States.

[e]Physics Department, Georgetown University, 37th and O Streets NW, Washington, D.C. 20057, United States

*Corresponding authors

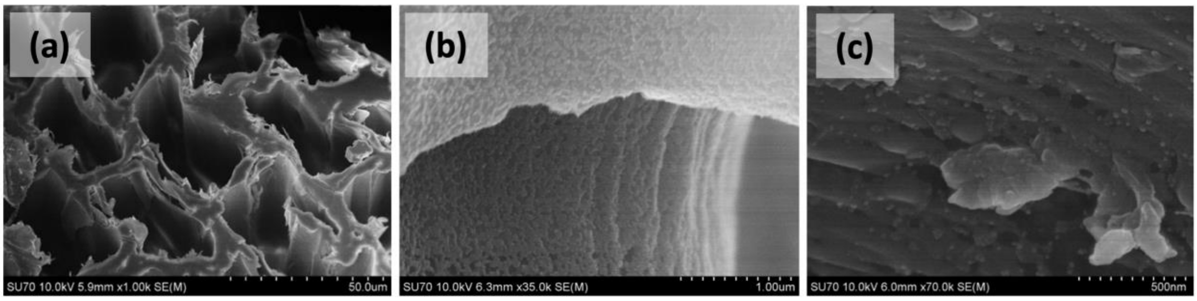

Figure S1. SEM images of the Fe_3O_4-modified Scots pine sapwood showing (a) unfilled cell lumina and ((b) and (c)) NP distribution in the pits (internal layer of wood block).

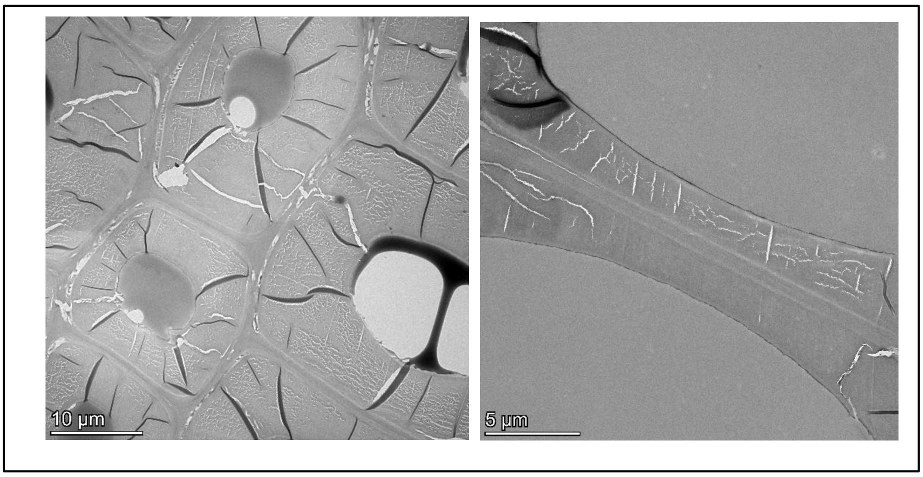

Figure S2. TEM images of the Fe_3O_4-modifed Scots pine showing cracks in the cell walls.

(a) 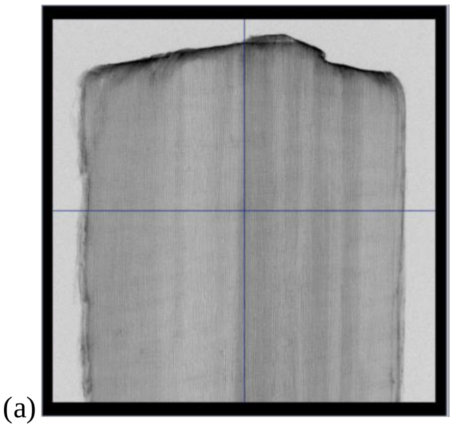 (b) 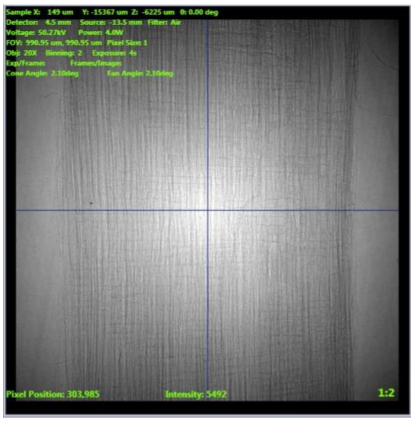

Figure S3. Micro-CT X-ray projection image (radiograph) of (a) the whole specimen of Fe_3O_4-treated Scots pine sapwood before cutting (Layer-2) showing greyish places of interest, and (b) image of the specimen used for CT analysis.

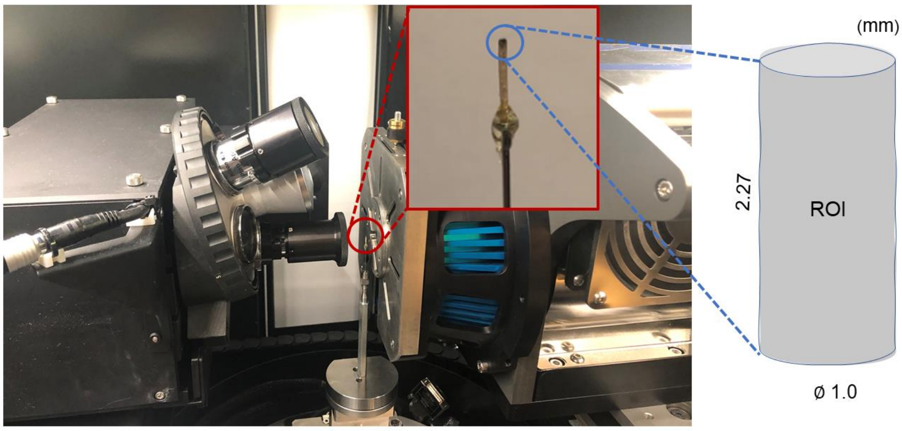

Figure S4. Micro-CT set up showing ROI (region of interest) and dimensions of the Fe_3O_4-NPs modified Scots pine used to study nanoparticle distribution over the entire specimen (photograph courtesy of Dr. Fredrik Forsberg, Luleå University of Technology, Sweden).

(a) 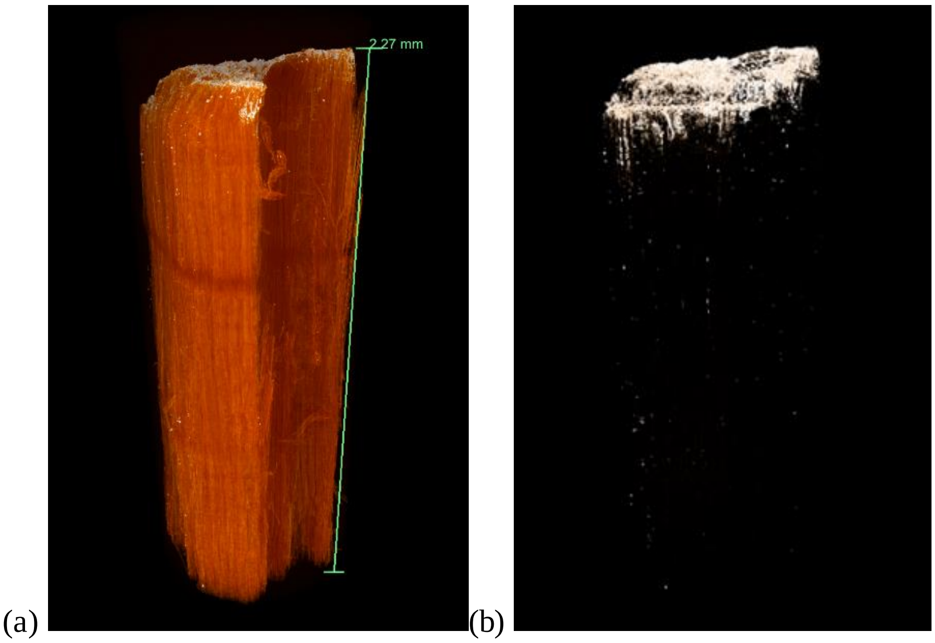

(b)

Figure S5. Reconstructed micro-CT 3D images of the Fe_3O_4-NPs modified wood showing (a) the whole scanned specimen that was cut from the internal part of the 1 cm × 0.5 cm × 0.5 cm wood block, and (b) high density profile with different distribution of the Fe_3O_4 NPs over entire specimen (bright places).

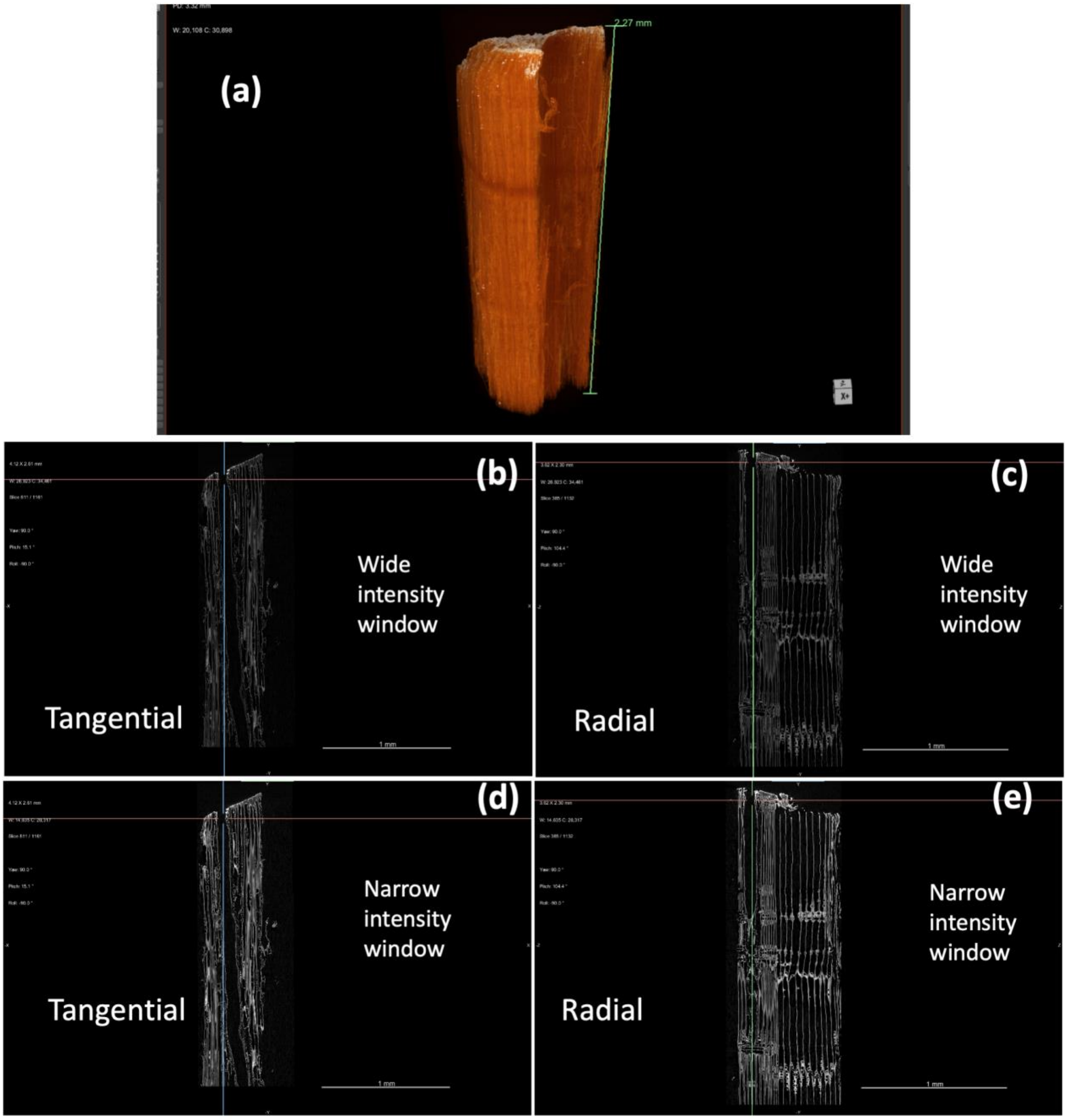

Figure S6. (a) Reconstructed micro-CT 3D image and X-ray projections of the Fe_3O_4-NPs modified wood from radial and tangential sections using different intensity windows: (b) and (c) – wide intensity window, (d) and (e) – narrow intensity window.

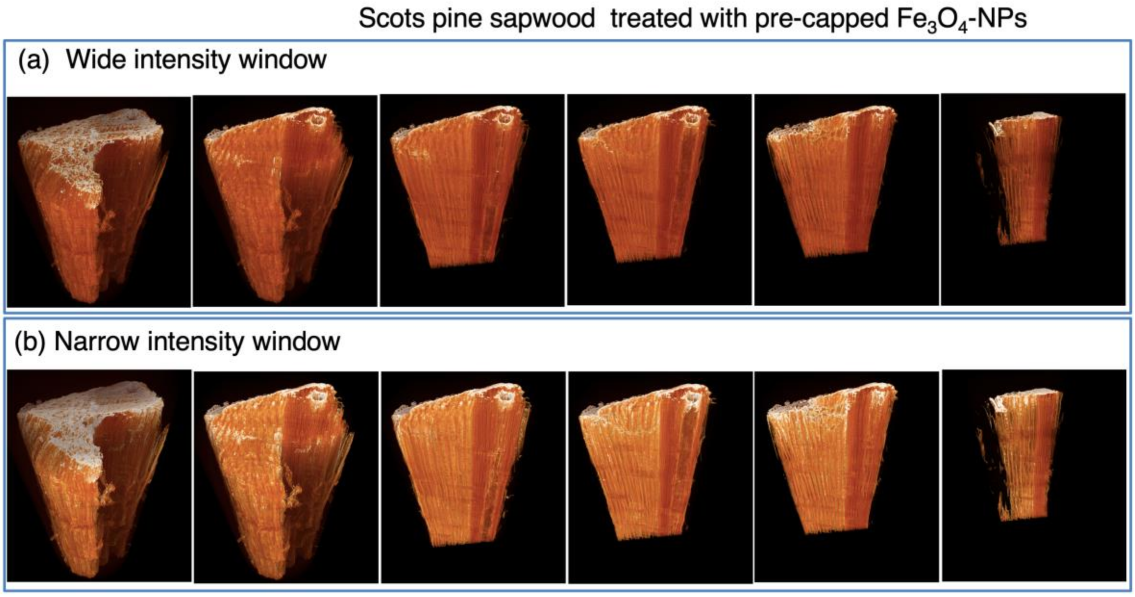

Figure S7. Reconstructed micro-CT images showing a number of the Fe_3O_4-NPs modified wood for two different intensity windows: (a) wide intensity window and (b) narrow intensity window.

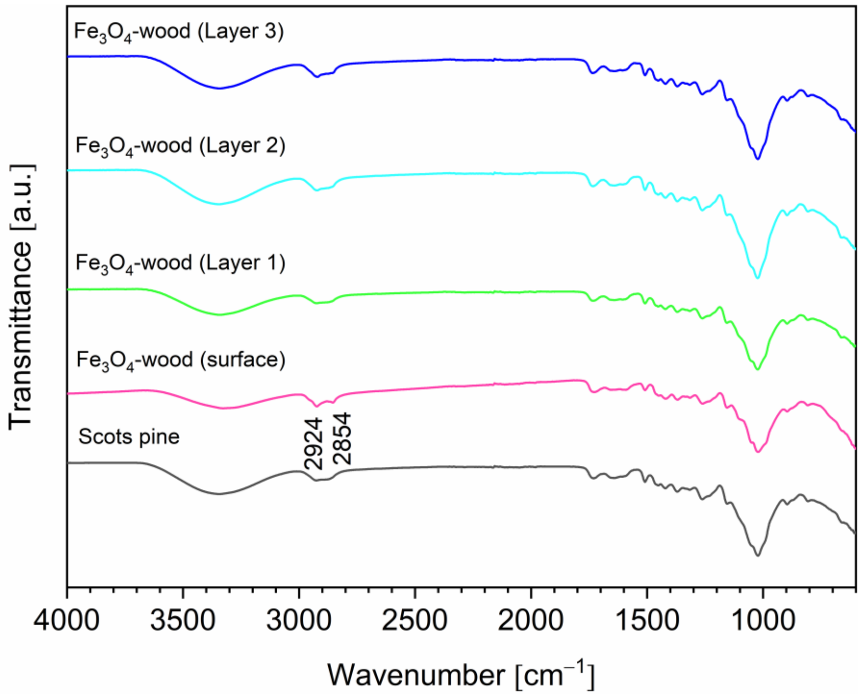

Figure S8. Full region of FTIR spectra of Scots pine sapwood and Fe_3O_4-modified wood.
